# Interaction of L1CAM with LC3 Is Required for L1-Dependent Neurite Outgrowth and Neuronal Survival

**DOI:** 10.3390/ijms241512531

**Published:** 2023-08-07

**Authors:** Gabriele Loers, Ralf Kleene, Viviana Granato, Ute Bork, Melitta Schachner

**Affiliations:** 1 Zentrum für Molekulare Neurobiologie, Universitätsklinikum Hamburg-Eppendorf, Martinistr. 52, 20246 Hamburg, Germany; 2 Department of Cell Biology and Neuroscience, Keck Center for Collaborative Neuroscience, Rutgers University, 604 Allison Road, Piscataway, NJ 08854, USA

**Keywords:** neural cell adhesion molecule L1, LC3, LIR motif, neurite outgrowth, neuronal survival

## Abstract

The neural cell adhesion molecule L1 (also called L1CAM or CD171) functions not only in cell migration, but also in cell survival, differentiation, myelination, neurite outgrowth, and signaling during nervous system development and in adults. The proteolytic cleavage of L1 in its extracellular domain generates soluble fragments which are shed into the extracellular space and transmembrane fragments that are internalized into the cell and transported to various organelles to regulate cellular functions. To identify novel intracellular interaction partners of L1, we searched for protein–protein interaction motifs and found two potential microtubule-associated protein 1 light-chain 3 (LC3)-interacting region (LIR) motifs within L1, one in its extracellular domain and one in its intracellular domain. By ELISA, immunoprecipitation, and proximity ligation assay using L1 mutant mice lacking the 70 kDa L1 fragment (L1-70), we showed that L1-70 interacts with LC3 via the extracellular LIR motif in the fourth fibronectin type III domain, but not by the motif in the intracellular domain. The disruption of the L1-LC3 interaction reduces L1-mediated neurite outgrowth and neuronal survival.

## 1. Introduction

Neural cell adhesion molecules are membrane proteins that play important roles in cell adhesion, migration, and signaling. One prominent member of this group is the surface glycoprotein L1, which is involved in cell migration, proliferation, survival and differentiation, axon fasciculation, and myelination during development of the nervous system, as well as in neurogenesis, neural plasticity, learning and memory, and regeneration in the adult nervous system [1,2,3,4,5,6,7]. In tumor types of different origins, L1 expression is linked to mobility of tumor cells, metastatic potential, malignancy, and drug resistance, and it is a marker for poor prognosis [8,9,10,11]. L1 also plays pro-angiogenic roles in endothelial cells of tumor-associated vessels [12]. L1 contains an extracellular N-terminal part with six immunoglobulin-like (Ig) domains and five fibronectin type III (FNIII) domains, a transmembrane domain, and a C-terminal intracellular domain [13,14]. In humans, mutations in the L1 gene cause the development of the L1 syndrome with varying severity. The L1 syndrome is characterized by X-linked hydrocephalus with stenosis of the aqueduct of Sylvius, mental retardation, aphasia, adducted thumbs, spastic paraplegia type 1, corpus callosum agenesis, and congenital aganglionic megacolon [9,11,15,16]. Moreover, impaired L1 functions are associated with neurological and psychiatric disorders, such as fetal alcohol syndrome, Hirschsprung’s disease, Alzheimer’s disease, schizophrenia, and autism [17,18,19,20,21,22,23]. In mice, mutations in L1 cause developmental defects of the nervous system and, depending on the mutation, the generation of an L1 syndrome-like phenotype [24,25,26]. L1-deficient mice show alterations in neuronal cell differentiation, migration and survival, axon outgrowth and fasciculation, synaptogenesis, myelination, synaptic plasticity, learning, memory, behavior, and regeneration after injury [7,27,28,29,30,31,32,33,34,35,36].

Proteolysis of full-length L1 was shown to be important for L1’s functions, and extracellular and intracellular L1 fragments were found in different locations and organelles and regulate different cellular functions [6,10,11,36,37,38,39,40,41,42,43,44,45,46]. A proteolytically active form of myelin basic protein generates a transmembrane C-terminal fragment of 70 kDa (L1-70) after the stimulation of L1 signal transduction, e.g., with function-triggering L1 antibody 557 [36,42]. L1-70 contributes to neuritogenesis, neuronal migration and survival, astrogliosis, gene expression, and mitochondrial homeostasis [24,36,40,41,42,47,48]. Of note, the upregulation of L1-70 expression in a mouse model of Alzheimer’s disease by parabiosis with a wild-type mouse reduced amyloid-β plaques, and mutant mice lacking L1-70 are abnormal in behavior [49,50].

To identify novel interaction partners of intracellular L1 fragments and to further determine the function of these L1 fragments, we searched for protein interaction motifs in L1 and intracellular proteins interacting with these motifs. We found two potential microtubule-associated protein 1 light-chain 3-interacting region (LIR) motifs in L1: one in the fourth FNIII domain and one in the intracellular domain. Proteins containing a LIR motif bind to the LIR docking site of proteins belonging to the autophagy-related protein 8 (ATG8) family. The microtubule-associated protein 1 light chain 3 alpha (MAP1LC3A; also called autophagy-related ubiquitin-like modifier LC3 A or LC3) is a mammalian homolog of ATG8E. The LC3 conjugation system is required for elongation and maturation of the autophagosome [51] and mitochondria and the endoplasmic reticulum function in the initiation of autophagy. ATG8/LC3 proteins are recruited to mitochondria, not only for the removal of mitochondria by mitophagy, but also to provide cellular anchorage and to share the lipid moieties required for elongating the initial phagophore [52,53]. Furthermore, ATG8/LC3 proteins are not only involved in autophagy, but also phagocytosis and endocytosis [54,55,56]. LC3-associated endocytosis has recently been identified and shown to regulate neuroinflammation and to play a role in neurodegeneration [54,55,57].

Here, we show by ELISA and proximity ligation assay that L1 interacts directly with LC3 and that this interaction is mediated by the LIR motif within the fourth FNIII domain, but not by the LIR motif in the intracellular domain. LC3 interacts mainly with L1-70 generated by myelin basic protein, as seen by a reduction of L1/LC3 interactions in non-stimulated cerebellar and cortical neurons from L1-70-lacking mutant mice, and L1 antibody-enhanced L1/LC3 interactions were not observed in L1-70-lacking neurons in contrast to wild-type neurons. The treatment of cultured cortical neurons with a cell-penetrating peptide containing the LIR motif of the fourth FNIII domain reduces the interaction as well as L1-dependent neurite outgrowth and neuronal cell survival.

## 2. Results

### 2.1. L1 Carries Putative LIR Motifs in Its Extracellular and Intracellular Domains

Previous research from our group showed that fragments of L1 are present inside of the cell in the cytosol, in nuclei, and in mitochondria, and that L1 regulates mitochondrial and nuclear functions [24,36,40,41,43,44,45,46,48]. Since only a few proteins binding to these intracellular L1 fragments are known, we set out to identify novel intracellular interaction partners. For this aim, we searched for protein–protein interaction motifs in the extracellular and intracellular L1 domains.

To search for putative LIR motifs in L1, we used the canonical LIR core motif [WFY]_0_-X_1_-X_2_-[LVI]_3_ [58] to scan the mouse, rat, human, and bovine L1 sequences, and identified 17–21 putative LIR motifs depending on the species. To refine the search, the so-called xLIR motif [ADEFGLPRSK]_-2_-[DEGMSTV]_-1_-[WFY]_0_-[DEILQTV]_1_-[ADEFHIKLMPST]_2_-[ILV]_3_, which is a redefined LIR motif [59], was used to scan L1 sequences for LIR motifs. Since one putative LIR motif (LSYQPL) was only found in the intracellular domain of bovine L1 at position 948–953, we used the less stringent xLIR motif [ADEFGLPRSK]_-2_-[DEGMSTV]_-1_-[WFY]_0_-[HKRN]_1_-[ADEFHIKLMPST]_2_-[ILV]_3_ with the conservative glutamine (Q) substitutions histidine (H), lysine (K), arginine (R), and asparagine (N) at position X_1_ and identified one motif in the fourth FNIII domain (_952_LSYHPV_957_) and one in the in the intracellular domain (_1177_GEYRSL_1182_) of mouse L1. These motifs were also found in the fourth FNIII domain and in the intracellular domains of rat (_952_LSYHPL_957_, _1176_GEYRSL_1181_), human (_953_LSYHPL_958_, _1174_GEYRSL_1179_), and bovine (_948_LSYQPL_953_, _1169_GEYRSL_1174_) L1. These findings raise the question of whether L1 directly interacts with LC3 and if this interaction is mediated by the LIR motifs in L1.

### 2.2. Mouse L1 Binds to LC3 via Its Extracellular LIR Motif LSYHPV in the Fourth FNIII Domain, but Not via Its Putative Intracellular LIR Motif GEYRSL

**Figure 1:**
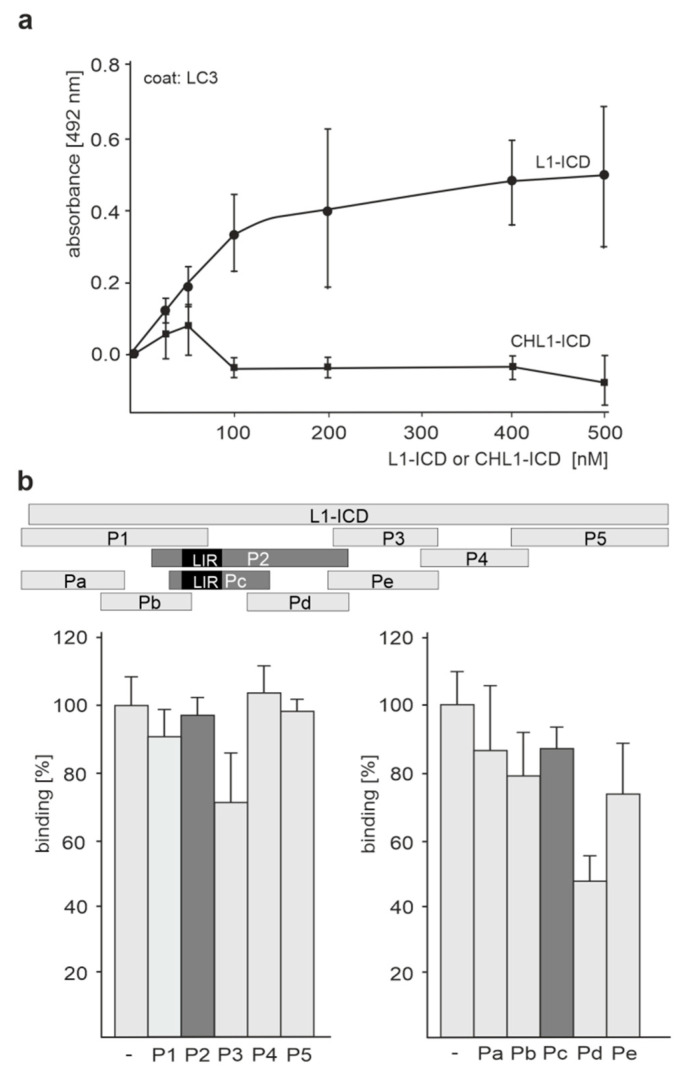
The direct interaction of L1-ICD with LC3 is not mediated by its putative intracellular LIR motif. (**a**) Recombinant LC3 was substrate-coated and incubated with increasing concentrations of L1-ICD or CHL1-ICD. (**b**) Recombinant LC3 was substrate-coated and incubated with L1-ICD in the absence (-) or presence of a 5-fold excess of L1 peptides P1, P2, P3, P4, or P5, which cover the entire L1-ICD sequence, or of a 5-fold excess of L1 peptides Pa, Pb, Pc, Pd, or Pe, which cover the N-terminal, membrane-proximal 73 amino acids of L1-ICD. The positions of the peptides in L1-ICD and the position of the intracellular LIR motif in P2 and Pc are shown. (**a**,**b**) Binding was determined by ELISA using mouse L1 antibody C-2 (**a**,**b**) or goat CHL1 antibody C-18 (**a**) in conjunction with horse radish peroxidase (HRP)-conjugated secondary antibodies. Mean values ± SD from three independent experiments carried out in triplicates are shown.

To investigate if L1 interacts with LC3 and if this binding is mediated by the LIR motif in the extracellular or intracellular L1 domain, an ELISA was performed. First, recombinant LC3 was used as a substrate-coat and was incubated with the recombinant His-tagged intracellular domain of L1 (L1-ICD). The intracellular domain of CHL1 (CHL1-ICD), the close homolog of L1, served as negative control. A concentration-dependent binding of the L1-ICD, but not of the CHL1-ICD to LC3, was observed (Figure 1a). Next, we used five peptides covering the 114 amino acids of the L1-ICD sequence for competition and observed that none of the peptides significantly inhibited the binding of L1-ICD to LC3 (Figure 1b). When four overlapping peptides which cover the 73 N-terminal amino acids of L1-ICD were used for competition ELISA, a slight reduction of the binding between L1-ICD and LC3 was only observed with peptide Pd comprising amino acids 40–57 of L1-ICD (Figure 1b). Since the LIR motif-containing peptides P2 and Pc did not reduce the binding of L1-ICD to LC3, we concluded that the putative LIR motif GEYRSL in the intracellular domain of L1 does not mediate the interaction of L1 with LC3.

**Figure 2:**
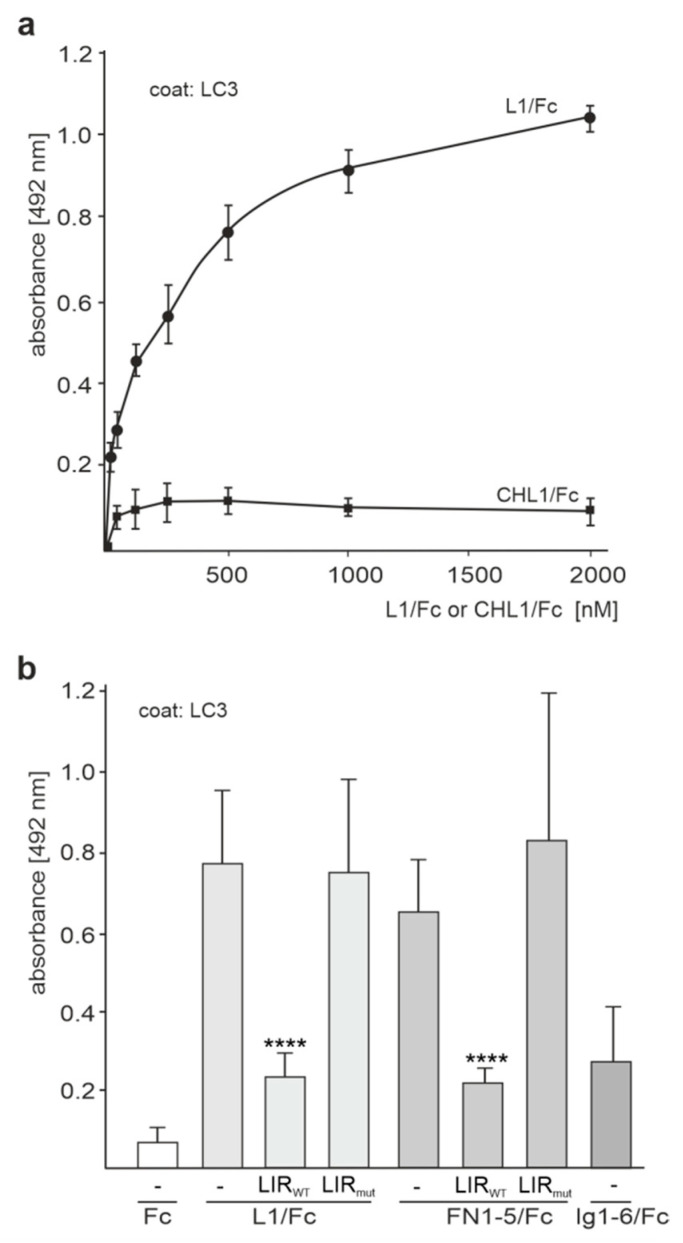
The direct interaction of L1 with LC3 is mediated by the LIR motif LSYHPV in the fourth FNIII domain. (**a**) Recombinant LC3 was substrate-coated and incubated with increasing concentrations of L1/Fc and CHL1/Fc. (**b**) Recombinant LC3 was substrate-coated and incubated with L1/Fc, Ig1–6/Fc, and FN1–5/Fc in the absence (-) or presence of a 5-fold excess of the LIR_WT_ or LIR_mut_ peptide. (**a**,**b**) Binding was determined by ELISA using HRP-conjugated anti-Fc antibody. Mean values ± SD from three independent experiments carried out in triplicates are shown. **** *p* < 0.001 relative to the treatment in the absence of peptides, one-way ANOVA with Bonferroni’s multiple comparison test.

Next, we investigated whether the putative LIR motif in the fourth FNIII domain could mediate the binding of full-length L1 or L1 fragments to LC3. To this aim, recombinant fusion proteins of the extracellular domains of L1 or CHL1 and Fc from human IgG (L1/Fc and CHL/1Fc, respectively) were tested by ELISA for binding to LC3. L1/Fc, but not CHL1/Fc, showed a concentration-dependent binding to LC3 (Figure 2a). Next, we used the LIR_WT_ peptide containing the extracellular LIR motif LSYHPV or the LIR_mut_ peptide containing the mutated LIR motif LSAHPA for competition ELISA with L1/Fc and fusion proteins containing human Fc and either FNIII domains 1–5 (FN1–5/Fc) or Ig-like domains 1–6 (Ig1–6/Fc). L1/Fc and FN1–5/Fc, but not Fc, bound to LC3, while Ig1–6/Fc showed only low binding to LC3. Moreover, the LIR_WT_ peptide reduced the binding of L1/Fc and FN1–5/Fc by 60–65%, whereas the LIR_mut_ peptide did not affect the binding of L1/Fc and FN1–5/Fc to LC3 (Figure 2b). These results indicate that the LIR motif in the fourth FNIII domain mediates the binding of the L1 to LC3.

### 2.3. LC3 Interacts with L1 in Cortical Neurons

**Figure 3:**
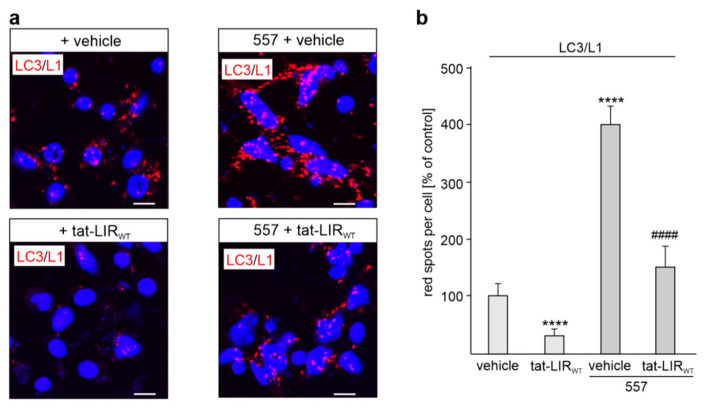
Interaction of L1 with LC3 in cultured cortical neurons is enhanced by function-triggering L1 antibody and reduced by a cell-penetrating peptide containing the extracellular LIR motif LSYHPV. Cultured cortical neurons were treated without (vehicle) or with the tat-LIR_WT_ peptide (tat-LIR_WT_) followed by treatment without (unstimulated) or with (stimulated) the function-triggering L1 antibody 557 (557). The neurons were fixed and then subjected to proximity ligation with mouse L1 antibody C-2 and a rabbit LC3 antibody. Nuclei are stained with DAPI (blue). (**a**) In the representative images, red spots indicate the close interaction of L1 and LC3. Scale bars: 10 μm. (**b**) Mean values + SEM are shown for the average numbers of L1/LC3-positive red spots per cell from three independent experiments (**** *p* < 0.001 relative to vehicle treatment; ^####^ *p* < 0.001 relative to 557 treatment; one-way ANOVA with Bonferroni’s multiple comparison test).

To substantiate the notion that L1 and LC3 interact directly and that this interaction is mediated by the LIR-motif in the fourth FNIII domain, proximity ligation assay was performed. This assay is a very sensitive method to detect protein interactions as red spots when the proteins are in proximity of 40 nm or less. We treated cortical neurons with the cell-penetrating tat-LIR_WT_ peptide in the absence or presence of the function-triggering L1 antibody 557 and then subjected the neurons to proximity ligation assay with a mouse L1 antibody and a rabbit LC3 antibody. L1 antibody 557 enhanced the number of red spots, which indicate L1/LC3 interactions, approximately four times, while the tat-LIR_WT_ peptide reduced the L1/LC3 interactions in non-stimulated and stimulated neurons by 69 ± 10% and 63 ± 10%, respectively (Figure 3a,b). This result shows that L1 and LC3 interact in a cellular context and verifies that the LIR motif LSYHPV in the fourth FNIII domain of L1 mediates this interaction.

### 2.4. LC3 Interacts with L1-70

**Figure 4:**
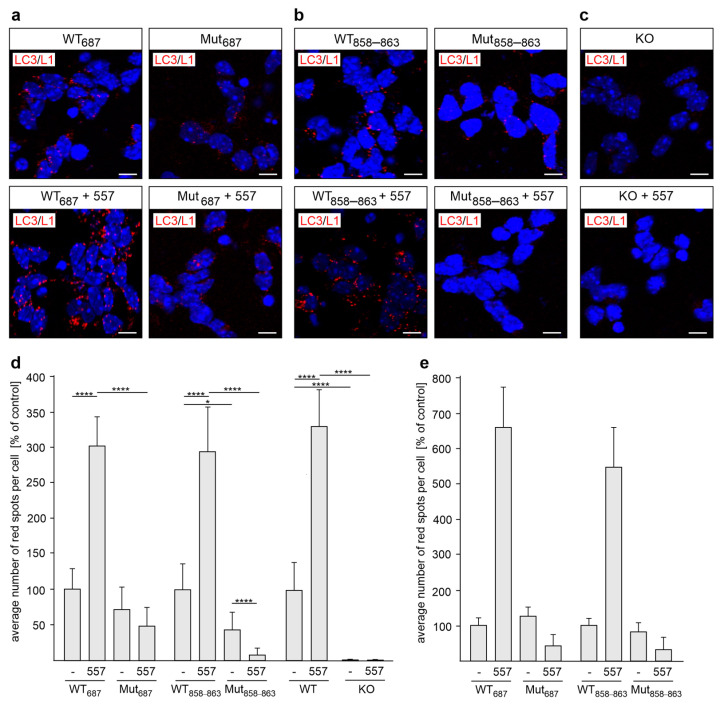
LC3 interacts with L1-70 in cultured cerebellar and cortical neurons. Cultured cerebellar (**a**–**d**) and cortical (**e**) neurons from L1-70-lacking L1_687_ (Mut_687_) or L1_858–863_ (Mut_858–863_) male mutant mice (**a**–**e**) or L1-deficient (KO) male mice (**d**) and their male wild-type (WT) littermates (**a**–**e**) were treated without (-) or with L1 antibody 557 (557). The neurons were fixed and subjected to proximity ligation with mouse L1 antibody C-2 and a rabbit LC3 antibody. Nuclei are stained with DAPI (blue). (**a**–**c**) In the representative images, red spots indicate a close interaction of L1 with LC3. Scale bars: 10 μm. (**d**,**e**) Mean values + SD from three independent experiments (**d**) or one experiment (**e**) are shown for the average numbers of L1/LC3-positive spots per cell in cerebellar (**d**) or cortical (**e**) neurons (* *p* < 0.05, **** *p* < 0.001 one-way ANOVA with Dunn’s multiple comparison test).

Since L1-70 was found in the cytosol, in mitochondria, and in nuclei [24,36,40,41,43,44,45,46,48] and interacts with NADH:ubiquinone oxidoreductase core subunit V2, topoisomerase I, and peroxisome proliferator-activated receptor γ, which play important functions in mitochondrial homeostasis and cellular health [48], this fragment might also interact with LC3 to regulate mitophagy or autophagy. To determine whether L1-70 interacts with LC3, cerebellar and cortical neurons from the L1-70-lacking L1_687_ and L1_858–863_ mutant mice were used for proximity ligation assay. The L1/LC3 interactions in non-stimulated mutant cerebellar neurons were slightly reduced in comparison to those in wild-type neurons (Figure 4a–e). After the stimulation of cultured neurons with L1 antibody 557, wild-type cerebellar neurons revealed an enhanced number of L1/LC3 interactions, whereas no enhanced number of L1/LC3 interactions was observed for L1-70-lacking L1_687_ and L1_858–863_ mutant neurons (Figure 4a–d). Almost no L1/LC3-positive spots were observed with L1-deficient cerebellar neurons (Figure 4d). Similar results were observed with wild-type and mutant cortical neurons: the numbers of L1/LC3 interactions in non-stimulated wild-type and mutant neurons were similar, while enhanced numbers of L1/LC3 interactions were observed in stimulated wild-type neurons but not in stimulated mutant neurons (Figure 4e). The combined results indicate that LC3 interacts with L1-70.

**Figure 5:**
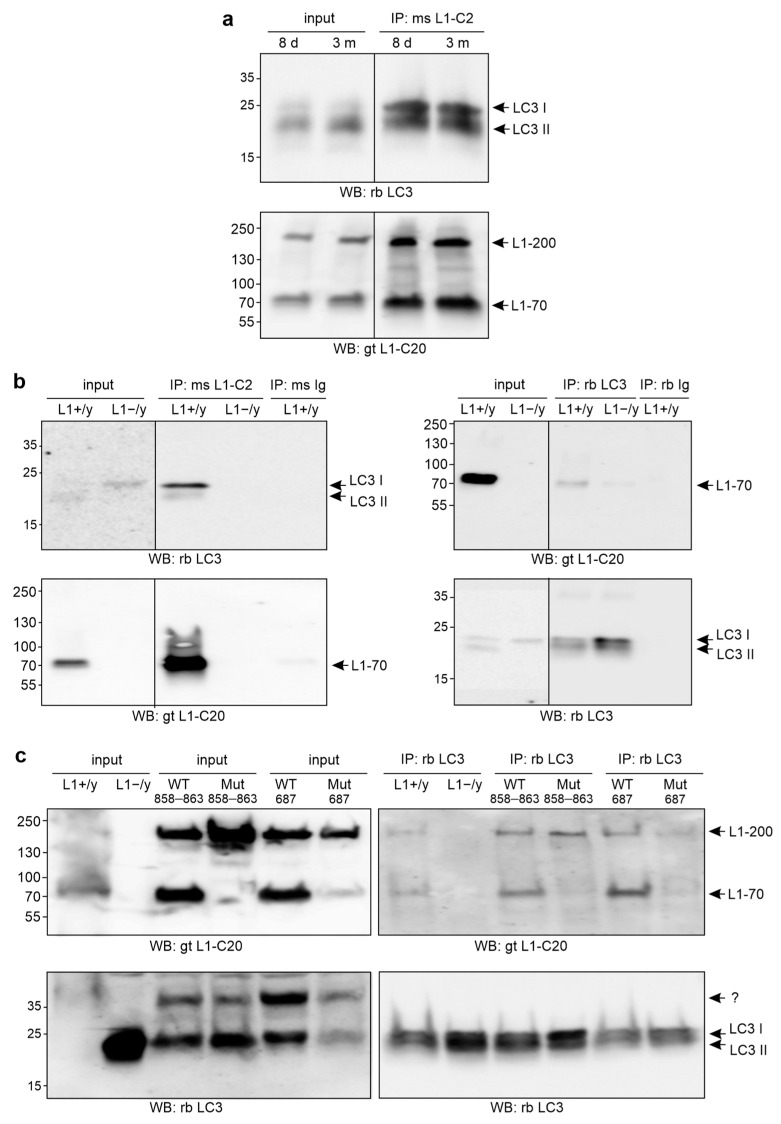
LC3 interacts with L1-70. Non-nuclear fractions from brains of postnatal and adult wild-type mice (**a**), from adult wild-type (L1+/y) and L1-deficient (L1−/y) male littermates (**b**,**c**), and from adult L1-70-lacking L1_687_ (Mut 687) and L1_858–863_ (Mut 858–863) mutant males and their wild-type male littermates (WT 687, WT 858–863) were used for immunoprecipitation (IP) with mouse L1 antibody C-2 (ms L1-C2) (**a**,**b**), non-immune control rabbit (rb Ig) or mouse (ms Ig) antibodies (**b**), or rabbit LC3 antibody (rb LC3) (**b**,**c**). (**a**–**c**) The non-nuclear fractions (input) and immunoprecipitates were subjected to Western blot (WB) analysis of the immunoprecipitates with goat L1 antibody C-20 (gt L1-C20) or rabbit LC3 antibody (rb LC3). (**a**,**b**) Lanes that were not adjacent to each other, but derived from the same blot or were exposed differently are indicated by vertical lines. The arrows indicate full-length L1 (L1-200), L1-70, LC3-I, and LC3-II. The question mark indicates a unknown LC3-positive band.

To further analyze the association of L1 with LC3 and to investigate development- and age-related differences in L1/LC3 interactions, we performed immunoprecipitation with immobilized L1 antibody and non-nuclear brain fractions from developing 8-day-old and young adult 3-month-old wild-type mice. This fraction contains mainly membrane proteins or membrane-associated proteins from the plasma membrane, mitochondria, endoplasmic reticulum, Golgi apparatus, and endosomes, but contains only very low amounts of soluble cytosolic and nuclear proteins. Western blot analysis with LC3 antibody revealed similar levels of LC3-immunopositive double bands in the L1 immunoprecipitates of the non-nuclear brain fractions from 8-day-old and 3-month-old wild-type mice (Figure 5a). The upper band of the LC3 double band corresponds to the soluble form of LC3 (LC3-I), while the lower band represents the membrane-bound form of LC3 (LC3-II) [60]. LC3-II is localized mainly on autophagosomes and autolysosomes and is generated by the lipidation of LC3-I during the formation of autophagosomes [60]. Detection with a goat L1 antibody showed that the immobilized L1 antibody immunoprecipitated equal amounts of full-length L1 and L1-70 from non-nuclear fractions of postnatal and adult mice (Figure 5a). Results indicate that L1 interacts with soluble and membrane-associated LC3 in the brains of early postnatal mice to a similar extent as in the brains of adult mice.

Next, we performed immunoprecipitation with immobilized L1, LC3 or control antibodies and a non-nuclear brain fraction from 8-month-old wild-type and L1-deficient mice. By Western blot analysis, L1-70, but not full-length L1, was detected in the LC3 immunoprecipitates from the non-nuclear wild-type brain fraction and a LC3-immunopositive double band with LC3-I as predominant LC3 form was seen in the L1 immunoprecipitates of this fraction (Figure 5b). These bands were hardly detectable in control immunoprecipitates using non-immune antibodies and in L1 and LC3 immunoprecipitates of the non-nuclear brain fractions from L1-deficient mice (Figure 5b). Detection with a goat L1 antibody showed L1-70 in the L1 immunoprecipitates from the wild-type brain fraction, but not from the L1-deficient brain fraction (Figure 5b), and detection with the LC3 antibody showed that similar amounts of LC3-I and LC3-II were immunoprecipitated from the non-nuclear wild-type and L1-deficient brain fractions by the immobilized LC3 antibody (Figure 5b).

To gain more evidence for the association of L1-70 with LC3, immunoprecipitations with immobilized L1, LC3, or control antibodies and non-nuclear brain fractions from 8-month-old L1-deficient and 3-month-old L1-70-lacking L1_687_ and L1_858–863_ mutant mice, as well as from age-matched wild-type littermates, were performed. By Western blot analysis, bands for L1-70 and full-length L1 were seen in the LC3 immunoprecipitates of the non-nuclear wild-type brain fractions, whereas full-length L1, but no or low amounts of L1-70, were found in the LC3 immunoprecipitates of the non-nuclear brain fractions from L1-deficient and L1-70-lacking mutant mice (Figure 5c). Detection with LC3 antibody showed that similar amounts of LC3 were immunoprecipitated from all fractions by the immobilized LC3 antibody. Results show that LC3 associates with L1-70. 

### 2.5. Disturbance of the L1/LC3 Interaction by the Cell-Penetrating Tat-LIR_WT_ Peptide Inhibits L1-Dependent Neurite Outgrowth and Neuronal Cell Survival

**Figure 6:**
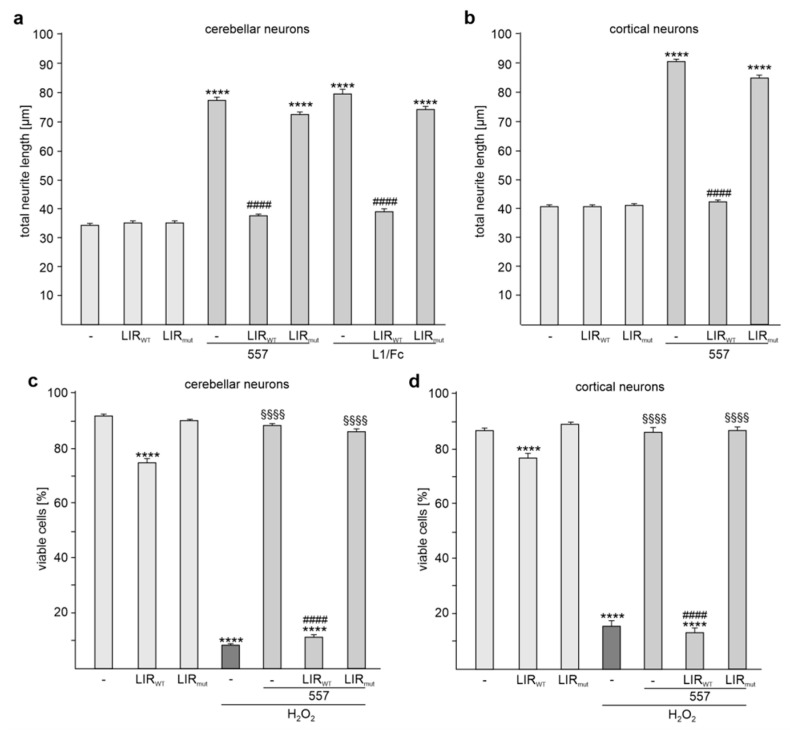
Disturbance of the L1/LC3 interaction inhibits L1-dependent neurite outgrowth and cell survival from cortical and cerebellar neurons. Cerebellar (**a**,**c**) and cortical (**b**,**d**) neurons were treated without (-) or with tat-LIR_WT_ or tat-LIR_mut_ peptide followed by treatment without or with L1 antibody 557 (557) (**a**–**d**) or L1/Fc (**a**). (**a**,**b**) Mean values + SEM from three independent experiments are shown for total neurite lengths (**** *p* < 0.001 relative to non-stimulated neurons in the absence of the peptides, ^####^ *p* < 0.001 relative to 557 antibody-stimulated neurons in the absence of the peptides; one-way ANOVA with Dunn’s multiple comparison test). (**c**,**d**) Mean values + SEM from three independent experiments are shown for the numbers of viable cells relative to total number of cells (**** *p* < 0.0001 relative to non-stimulated neurons in absence of peptides and H_2_O_2_, ^####^ *p* < 0.0001 relative to stimulated neurons in the absence of peptides and the presence of H_2_O_2_, ^§§§§^ *p* < 0.0001 relative to non-stimulated neurons in the absence of peptides and the presence of H_2_O_2_; one-way ANOVA with Tukey’s multiple comparison test).

To determine whether the interaction of LC3 and L1 is important for L1 functions, we treated neurons with or without the tat-LIR_WT_ peptide and with or without function-triggering L1 antibody 557 and determined neurite outgrowth and neuronal survival. In the absence of the L1 antibody 557, the tat-LIR_WT_ peptide did not affect neurite outgrowth from cerebellar and cortical neurons, while this peptide reduced L1 antibody 557-enhanced neurite outgrowth (Figure 6a,b). However, the tat-LIR_mut_ peptide with a mutated LIR motif did not reduce the antibody 557-enhanced neurite outgrowth (Figure 6a,b). It is noteworthy to mention that the tat-LIR_WT_ peptide, but not the tat-LIR_mut_ peptide, also reduced the L1/Fc-enhanced neurite outgrowth from cerebellar neurons (Figure 6a). 

Next, we investigated whether the tat-LIR_WT_ peptide has an effect on L1-dependent neuronal survival. Hydrogen peroxide-induced cell death was reduced in the presence of L1 antibody 557 (Figure 6c,d). The tat-LIR_WT_ peptide, but not the tat-LIR_mut_ peptide, reduced the antibody 557-induced cell survival of cerebellar and cortical neurons (Figure 6c,d). In the absence of L1 antibody 557, hydrogen peroxide-induced cell death was not altered by the tat-LIR_WT_ peptide alone (Figure 6c,d). 

Results indicate that L1-dependent neurite outgrowth and neuronal survival depends on the interaction of L1 with LC3 and that disturbance of this interaction reduces L1-stimulated neurite outgrowth and cell survival.

## 3. Discussion

Here we identified putative LIR motifs in the extracellular and intracellular domain of L1. The extracellular LIR motif LSY[QH]P[LV] in the fourth FNIII domain and the intracellular LIR motif GEYRSL were found in mouse, rat, human, and bovine L1, suggesting that these putative motifs are evolutionarily highly conserved in L1 of all mammalian species.

ELISA showed that the recombinant L1-ICD binds to LC3. Recombinant fusion proteins of Fc and extracellular L1 domains bind to LC3 when the fusion protein contains the fourth FNIII domain. The binding of the intracellular L1 domain to LC3 is not mediated by its LIR domain GEYRSL, since the binding was not affected by peptides containing this LIR motif. In contrast, the binding between LC3 and extracellular L1 domains that contain the fourth FNIII domain is mediated by the extracellular LIR motif LSY[QH]P[LV] in the fourth FNIII domain, since this binding was reduced by a peptide containing this LIR motif. It is conceivable that the binding of the L1-ICD to LC3 is mediated either by at least two distal sequence stretches in the L1-ICD or by the secondary/tertiary structure of the L1-ICD.

Immunoprecipitation indicates that LC3 associates mainly with L1-70, which was not detectable in LC3 immunoprecipitates from non-nuclear brain fractions of L1-70-lacking mutant mice. Of note, full-length L1 was also observed in the LC3 immunoprecipitates of wild-type and L1-70-lacking mutant mice. We propose that LC3 also interacts with full-length L1, most likely not via its extracellular LIR motif but via its intracellular domain.

After its generation at the plasma membrane, L1-70 is transported from the cytoplasm into nuclei and mitochondria and is also present at the mitochondrial surface [40]. L1-70 is crucial for neurite outgrowth, neuronal migration, neuronal survival, and mitochondrial homeostasis [24,36,40,41,42,43,47]. Furthermore, mice lacking L1-70 display a reduced mitochondrial membrane potential and lower ATP levels [24]. Since L1-70 contains the LIR motif-containing fourth FNIII domain and is present in the cytoplasm and at the cytoplasmic surface of mitochondria, it is plausible that the interaction of L1-70 with LC3 is involved in autophagy and/or mitophagy, the selective autophagy of mitochondria. L1’s extracellular LIR motif LSY[QH]P[LV] has a tyrosine (Y) at the absolutely conserved aromatic [WFY]_0_ position. LIR motifs with tyrosine at position X_0_ are less abundant than LIR motifs with tryptophan (W) and phenylalanine (F) at that position: out of 100 LIR motifs only 10 contain Y at the [WFY]_0_ position, while 42 and 48 contain W or F at this position, respectively [58]. Among proteins carrying LIR motifs with Y at position X_0_ are mitochondrial LC3 receptors, which are involved in autophagy and/or mitophagy: FUN14 Domain Containing 1 (FUNDC1), Autophagy Related 4B (ATG4B), Mitogen-Activated Protein Kinase 15 (MAPK15) and Neighbor of BRCA1 Gene 1 Protein (NBR1). The mitochondrial outer membrane protein FUNDC1 binds to LC3 through its LIR motif DSYEVL and acts as an activator of hypoxia-induced mitophagy, ATG4B with its LIR motif LTYDTL is a cysteine peptidase which is required for autophagy as well as for mitophagy, MAPK15 carrying the LIR motif RVYQMI is an atypical MAPK protein that regulates basal and starvation-induced autophagy in a kinase activity-dependent manner, and NBR1 with the LIR motif EDYIII is an autophagy cargo receptor. The phosphorylation of tyrosine (Tyr-18) at position X_0_ in the LIR motif of FUNDC1 inhibits the activation of mitophagy, while hypoxia-induced dephosphorylation of this tyrosine and phosphorylation of the preceding serine at position X_-1_ (Ser-17) stabilizes the interaction of FUNDC1 with LC3 and triggers mitophagy [61].

Interestingly, the phosphorylation of serine (Ser-17) at position X_-1_ in the LIR motif GSWVEL of the mitochondrial LC3 receptor Bcl2 interacting protein 3 (BNIP3) promotes the interaction between BNIP3 and LC3 and induces mitophagy under oxidative stress conditions [62].

We identified a potential site for protein kinase C phosphorylation of serine in the extracellular LIR motif-carrying L1 sequence GYLLSYHPV (prediction score: 0.669). Thus, serine at position X_-1_ of L1’s extracellular LIR motif may become phosphorylated. We propose that L1 is a mitochondrial LC3 receptor like FUNDC1 and BNIP3 [63] and that the phosphorylation of serine in L1’s extracellular LIR motif at position X_-1_ promotes mitophagy, as seen for the phosphorylation of serine at position X_-1_ in the LIR motifs of FUNDC1 and BNIP3. It is conceivable that the binding affinity of LIR motifs to LC3 in general is regulated through phosphorylation (for review and references, see [64]).

L1 binds to dynamin-related protein 1 (DRP1) [40], which is a mitochondrial fission protein involved in the fragmentation of the mitochondrial network and the engulfment of damaged mitochondria. BNIP3 increases the association of DRP1 with mitochondria and promotes the selective mitophagy of small, depolarized mitochondria. Since DRP1 is required for Parkin-independent mitochondrial autophagy [65] and promotes mitochondrial homeostasis in the brain, we propose that L1, like BNIP3, is involved in Parkin-independent DRP1-mediated mitophagy.

Since L1-stimulated neurite outgrowth and neuronal survival were abrogated in the presence of a peptide containing the LIR motif of the fourth FNIII domain and basal cell survival was also reduced in the presence of this peptide, it is plausible that L1-stimulated neurite outgrowth relies on the interaction of L1 with LC3 and that interference with the L1/LC3 interaction leads to enhanced neuronal cell death. Furthermore, one may conclude that neurite outgrowth and the protection of neurons against oxidative stress depends on mitophagy that is regulated by the interplay of LC3 with L1. The disturbance of this interaction may have severe functional consequences and may lead to the development of neurodegenerative diseases or L1-associated diseases, such as the L1 syndrome. Of note, T to G mutation in exon 21 of the human L1 gene (c.2858T > G) results in a leucine to arginine exchange at position 953 in the extracellular LIR motif _953_LSYHPL_958_. This mutation causes symptoms of the L1 syndrome, e.g., adducted thumbs, hydrocephalus, and spastic paraplegia [66].

In summary, the extracellular LIR motif in the fourth FNIII domain of L1 is crucial for L1’s functions in the mouse brain.

## 4. Materials and Methods

### 4.1. Animals

L1-deficient mice [30] and gene-edited mice expressing L1 with arginine-to-alanine exchange at position 687 in the first FNIII domain (L1_687_ mutant) or with a mutation of the dibasic sequence RKHSKR to SKHSSS at positions 858–863 in the third FNIII domain (L1_858–863_ mutant) [39] have been described. Mice were bred and maintained at the Universitätsklinikum Hamburg-Eppendorf at 25 °C on a 12 h light/12 h dark cycle with ad libitum access to food and water. C57Bl/6J males and females, as well as L1_687_ mutant males, L1_858–863_ mutant males, L1-deficient males, and their wild-type male littermates, were used for all experiments. All animal experiments were conducted in accordance with the German and European Community laws on the protection of experimental animals and approved by the local authorities of the State of Hamburg (animal permit numbers ORG 1022, approval date 31 July 2020 and N116/2021, approval date 08 October 2021). The manuscript was prepared following the ARRIVE guidelines for animal research [67].

### 4.2. Reagents and Antibodies

Rabbit LC3 antibody (14600-1-AP; no RRID available) was from Proteintech Europe (Manchester, UK). Mouse L1 antibody C-2 (NCAM-L1; sc-514360; no RRID available) against the L1-ICD, goat L1 antibody C-20 (sc-1508, RRID:AB_631086), and goat CHL1 antibody C-18 (sc-34986, RRID:AB_1121563) were from Santa Cruz Biotechnology (Dallas, TX, USA). HRP-coupled goat anti-Fc antibody (109-035-190; RRID:AB_2888996) was from Jackson ImmunoResearch (Cambridgeshire, United Kingdom). Rat monoclonal function-triggering L1 antibody 557 has been described [68]. Secondary antibodies were from Dianova (Hamburg, Germany). Production and purification of recombinant L1/Fc containing the extracellular L1 domains (L1CAM_MOUSE, P11627; amino acids 35–1112), Ig1–6/Fc containing Ig1–6 domains of L1 (L1CAM_MOUSE, P11627; amino acids 35–600), FN1–5/Fc containing FNIII domains 1–5 of L1 (L1CAM_MOUSE, P11627; amino acids 613–1112), and CHL1/Fc containing the extracellular domains of the close homolog of L1 (CHL1) (NCHL1_MOUSE, P70232; amino acids 35–1015) have been described [69]. The production and purification of recombinant His-tagged L1 in the intracellular domain (L1-ICD) (L1CAM_MOUSE, P11627; amino acids 1147–1260) and CHL1 in the intracellular domain (CHL1-ICD) (NCHL1_MOUSE, P 70232; amino acids 1070–1186) were performed as described [39]. Recombinant human LC3B (UBI-60-0112-500) was from BIOZOL (Eching, Germany). Synthetic peptide P1 (CFIKRSKGGKYSVKDKEDTQVDSEARPMKDETFGE), P2 (RPMKDETFGEYRSLESDNEEKAFGSSQPSLNGDIK), P3 (GDIKPLGSDDSLADYGGSVD), P4 (CSVDVQFNEDGSFIGQYSGK), P5 (CSGKKEKEAAGGNDSSGATSPINPAVALE), Pa (FIKRSKGGKYSVKDKEDTQ), Pb (EDTQVDSEARPMKDET), Pc (KDETFGEYRSLES-DNEEK), Pd (NEEKAFGSSQPSLNGDIK), Pe (GDIKPLGSDDSLADYGGSVD), LIR_WT_ (SHNGVLTGYLLSYHPVEGESKEQ), and LIR_mut_ (SHNGVLTGYLLSAHPAEGESKEQ), as well as tat-LIR_WT_ (YGRKKRRQRRRSHNGVLTGYLLSYHPVEGESKEQ) and tat-LIR_mut_ (YGRKKRRQRRRSHNGVLTGYLLSAHPAEGESKEQ), both containing the cell-penetrating HIV tat sequence YGRKKRRQRRR [70], were from Schafer-N (Copenhagen, Denmark).

### 4.3. Motif Search

The Expasy ScanProsite tool (https://prosite.expasy.org/scanprosite/; accessed on 10 August 2021) was used for scanning the sequences of mouse (L1CAM_mouse), rat (L1CAM_rat), human (L1CAM_human), and bovine (E1BBI3_BOVIN) L1 for putative LIR motifs. NetPhos prediction tool (https://services.healthtech.dtu.dk/service.php?NetPhos-3.1; accessed on 11 November 2021) was used to identify phosphorylation sites in L1.

### 4.4. ELISA

For ELISA, 25 µL of 10 µg/mL recombinant LC3B were incubated overnight at 4 °C in 384-well microtiter plates with a high binding surface (Corning, Tewksbury, MA, USA). All the following steps were performed at room temperature. Wells were washed with Dulbecco’s phosphate-buffered saline with MgCl_2_ and CaCl_2_ (Sigma-Aldrich, Taufkirchen, Germany; D8662) (PBS), treated with blocking solution (2% essentially fatty acid-free bovine serum albumin in PBS) for 2  h, washed again with PBS containing 0.005% Tween 20 (PBST), and incubated with increasing concentrations of recombinant His-tagged L1-ICD and CHL1-ICD or L1/Fc and CHL1/Fc as ligands for 1 h under gentle agitation. For competition ELISA, 2.5 µM L1-ICD, L1/Fc, Ig1–6/Fc, or FN1–5/Fc were preincubated for 1 h without or with a 5-fold molar excess of peptide P1, P2, P3, P4, P5, Pa, Pb, Pc, Pd, Pe, LIR_WT_, or LIR_mut_. The mixtures were then incubated with substrate-coated recombinant LC3B. After washing two times with PBS and three times with PBST, L1 antibody C-2 (1:500), CHL1 antibody, or HRP-coupled anti-Fc antibody (1:2000) in blocking solution was applied for 1 h. When samples were incubated with L1 and CHL1 antibody they were washed two times with PBS and three times with PBST and then incubated with HRP-coupled anti-mouse and anti-goat antibody (diluted 1:2000 in blocking solution) for 1 h. All samples were washed two times with PBS and three times with PBST, and wells were incubated with 1 mg/mL ortho-phenylenediamine dihydrochloride (Thermo Fisher Scientific, Darmstadt, Germany) to detect bound proteins. The reaction was terminated by the addition of 25 µL 2.5 M sulphuric acid. Absorbance was measured at 492  nm with an ELISA reader (µQuant; BioTek, Bad Friedrichshall, Germany).

### 4.5. Cultures of Cortical and Cerebellar Neurons

Cortical neurons were prepared from cerebral cortices of 15.5- to 16.5-day-old embryos. Dissected cerebral cortices were incubated in Hanks’ balanced salt solution (HBSS) containing 0.025% trypsin (Sigma-Aldrich) at 37 °C for 30 min. The cortices were then incubated in HBSS containing 1% BSA (Sigma-Aldrich) and 1% trypsin inhibitor (T-6522, Sigma-Aldrich) at 37 °C for 2 min. After washing in HBSS, the tissue was mechanically dissociated, and the dissociated cells were cultured in Neurobasal medium (Thermo Fisher Scientific) supplemented with 1% B27, 2 mM L-glutamine, 100 U/mL penicillin, and 100 μg/mL streptomycin (all from Thermo Fisher Scientific). For the determination of neurite outgrowth, cells were seeded at a density of 5 × 10^4^ cells per well of a 48-well plate coated with poly-L-lysine (Sigma-Aldrich). For proximity ligation assay, cells were seeded onto 12 mm glass coverslips poly-L-lysine-coated in a 24-well plate at a density of 1.25 × 10^5^ cells per well. For cell death analysis, cells were seeded onto poly-L-lysine-coated 48-well plates at a density of 2.5 × 10^5^ cells per well.

Cerebellar neurons were prepared from the cerebella of 6- to 8-day-old mice. Cerebella were incubated with 10 mg/mL trypsin and 0.5 mg/mL DNase I (Sigma-Aldrich) in HBSS for 15 min at 37 °C, washed with HBSS, dissociated, and centrifuged at 100 × g for 15 min. Cells were then diluted in Neurobasal A medium (Thermo Fisher Scientific) supplemented with 2 mM L-glutamine (Thermo Fisher Scientific), 4 nM L-thyroxine (Sigma-Aldrich), 0.1 mg/mL BSA (Sigma-Aldrich), 12.5 μg/mL insulin (Sigma-Aldrich), 30 nM sodium selenite (Sigma-Aldrich), 100 μg/mL transferrin (Merck, Darmstadt, Germany), 0.1 mg/mL streptomycin, and 100 U/mL penicillin (Thermo Fisher Scientific). For the proximity ligation assay, cells were seeded onto poly-L-lysine-coated 12 mm glass coverslips in a 24-well plate at a density of 2.5 × 10^5^ cells per well. For the determination of neurite outgrowth, cells were seeded at a density of 5 × 10^4^ cells per well of a 48-well plate coated with poly-L-lysine (Sigma-Aldrich), and for neuronal survival analysis cells were seeded at a density 1.25 × 10^5^ cells per well of a 48-well plate coated with poly-L-lysine.

### 4.6. Proximity Ligation Assay

After seeding, cells were treated with vehicle solution or tat-LIR_WT_ peptide (100 µg/mL in vehicle solution) for 16–24 h. Following that, cells were treated with PBS (unstimulated) or L1 antibody 557 (50 µg/mL; stimulated) for 2 h. Cultured cells were fixed for 15–30 min at room temperature in 4% formaldehyde, washed with PBS, and subjected to proximity ligation assay using Duolink PLA products according to the manufacturer’s protocol (Sigma-Aldrich; Duolink PLA technology) with minor modifications. Cells were incubated with 1% Triton X-100 in PBS for 30 min, washed once with PBS, and blocked with Duolink Blocking solution for 30 min and incubated for 24 h at 4 °C with mouse L1 antibody C-2 (1:10 dilution) and rabbit antibody against LC3 (1:30 dilution) in Duolink Antibody Diluent. Cells were washed two times using Duolink Wash Buffer A and incubated with a mixture of secondary antibodies conjugated with oligonucleotides (Duolink PLA Anti-Rabbit Probe MINUS and Duolink Anti-Mouse PLA Probe PLUS). The proximity ligation reaction was then performed according to the manufacturer’s protocol using the Duolink In Situ Detection Reagent RED. Thereafter, the coverslips were incubated in 5 µg DAPI/mL PBS for 15 min, washed twice with PBS and mounted in Immuno-Mount (Thermo Fisher Scientific). Ten images per condition were taken using an Olympus F1000 confocal microscope (Evident, Hamburg, Germany) and analyzed using ImageJ software (ImageJ, version 1.53; https://imagej.nih.gov/ij/index.html; RRID:SCR_003070; accessed on 8 March 2022). Numbers of red dots and numbers of DAPI-stained nuclei were determined using ImageJ and the number of red dots per image was divided by the number of nuclei per image. The average values of red dots per cell (=nucleus) were determined in 10 images per condition.

### 4.7. Determination of Neurite Outgrowth and Neuronal Survival

To determine neurite outgrowth, cells were treated with cell-penetrating tat-LIR_WT_ or tat-LIR_mut_ peptides (50 µg/mL) and 557 antibody (50 µg/mL) 30 min after seeding. After 24 h in culture, cells were washed gently with pre-warmed culture medium, fixed in 2.5% glutaraldehyde for 30 min at room temperature, and stained with 1% toluidine blue and 1% methylene blue in 1% sodium tetraborate for 30 min at room temperature. Neurite outgrowth was analyzed by measuring the total length of neurites with a Zeiss AxioObserver.A1 microscope with the AxioVision 4.6 imaging system (Carl Zeiss, Oberkochen, Germany). For neurite outgrowth analysis for each condition, at least 100 neurons were counted.

To determine cell death, neurons were maintained overnight in serum-free medium and then treated with cell-penetrating tat-LIR_WT_ or tat-LIR_mut_ peptides (50 µg/mL) and L1 antibody 557 (50 µg/mL) and exposed to oxidative stress by the addition of 10 µM H_2_O_2_ for 24 h. Live and dead cells were then stained with calcein-AM (Thermo Fisher Scientific) and propidium iodide (Sigma-Aldrich) and imaged with a Zeiss AxioObserver.A1 microscope (Carl Zeiss) with a 20× objective (aperture 0.4) and the AxioVision 4.6 software (Carl Zeiss). Live and dead cells were counted in five images (containing 350–400 cells each) from each of the three wells per condition and experiment using ImageJ.

### 4.8. Immunorecipitation and Western Blot Analysis

For the preparation of nuclear and non-nuclear fractions, the Subcellular Protein Fractionation Kit for Tissue (Thermo Fisher Scientific) was used. Fractions in cytoplasmic extraction buffer (CEB) and membrane extraction buffer (MEB) were pooled and taken as non-nuclear fractions. Fractions in nuclear extraction buffer (NEB) were taken as nuclear fractions. Nuclear and non-nuclear fractions were used for immunoprecipitation.

For immunoprecipitation, Protein G magnetic beads (25 μL per sample) were washed twice in PBS, pH 7.4, and incubated in dilution buffer (1 mg/mL bovine serum albumin in PBS) for 10 min at 4 °C under rotation. The mouse L1 antibody C-2, rabbit LC3 antibody, or non-immune control rabbit or mouse antibodies (10 μg per sample) were diluted in dilution buffer. The diluted antibody solutions were incubated with the beads for 1 h at 4 °C under rotation. The beads were washed in dilution buffer for 5 min at 4 °C under rotation. A freshly prepared 13 mg/mL stock solution of dimethyl pimelimidate (DMP) was 1:1 diluted with wash buffer (0.2 M triethanolamine in PBS). After washing the beads in PBS, the diluted DMP solution (pH 8–9) was added to the beads, and beads were incubated for 30 min at room temperature (20–24 °C) under rotation. The beads were washed in wash buffer for 5 min at room temperature under rotation. The beads were then incubated in DMP solution and wash buffer for two further times. Finally, the beads were incubated twice in quenching buffer (50 mM ethanolamine in PBS) for 5 min at room temperature under rotation. After washing, the beads were incubated with brain fractions overnight at 4 °C under rotation. The beads were washed twice with lysis buffer and once with PBS. The beads were then boiled for 5 min in sample buffer (60 mM Tris-HCl, pH 6.8, 2% SDS, 1% β-mercaptoethanol, 6% glycerol, and 0.01% bromophenol blue).

For Western blot analysis, samples were run on 4–20% Mini-PROTEAN^®^ TGX™ Precast Protein Gels (BioRad, Feldkirchen, Germany). PageRuler™ Plus Prestained Protein Ladder (Thermo Fisher Scientific) was used as molecular weight marker. Proteins were then transferred to 0.45 μm Protran™ nitrocellulose membranes (VWR, Darmstadt, Germany) and stained with Ponceau S to control protein loading. The membranes were then incubated for 1 h in blocking solution (5% non-fat dry milk powder in Tris-buffered saline (TBS) (TBS; 10 mM Tris-HCl, pH 7.4; 150 mM NaCl) with 0.05% Tween 20 (TBST) and then incubated overnight with goat L1 antibody C-20 or rabbit LC3 antibody (1:1000) in blocking solution at 4 °C with shaking. After washing five times for 5 min in TBST, the membranes were incubated for 1 h with HRP-conjugated secondary anti-goat or anti-rabbit antibodies (1:20,000 in blocking solution). Bands were detected using enhanced chemiluminescent solution (ECL Prime and Select Western blotting reagents; GE Healthcare, Solingen, Germany) and a CCD camera (ImageQuant LAS-4000 mini; GE Healthcare).

## 5. Conclusions

Here, we show that L1-70 interacts with LC3 via the extracellular LIR motif in the fourth fibronectin type III domain and that this interaction is required for L1-mediated neurite outgrowth and neuronal survival. Phosphorylation at the serine residue in L1’s extracellular LIR motif might alter binding affinity to LC3. Since L1 also interacts with the mitochondrial fission protein DRP1 that is involved in mitophagy, we propose that L1, together with LC3, is involved in mitophagy. Altogether, we provide evidence that the extracellular LIR motif in the fourth FNIII domain of L1 contributes to L1’s functions in the nervous system and that impaired L1/LC3 interactions can lead to neurodevelopmental and neurodegenerative diseases.

## Data Availability

Not applicable.
